# Bifunctional Metamaterials Using Spatial Phase Gradient Architectures: Generalized Reflection and Refraction Considerations

**DOI:** 10.3390/ma14092201

**Published:** 2021-04-25

**Authors:** Octavian Danila, Doina Manaila-Maximean

**Affiliations:** Physics Department, ‘Politehnica’ University of Bucharest, 060042 Bucharest, Romania; doina.manaila@upb.ro

**Keywords:** metasurfaces, generalized reflection, generalized refraction, spatial phase gradient, polarization control, optical field guiding

## Abstract

We report the possibility of achieving normal-incidence transmission at non-normal incidence angles using thin interfaces made of metasurface structures with an appropriately-designed positive spatial phase distributions. The reported effect represents a consequence of generalized reflection and refraction, which, although having been studied for discovering exotic effects such as negative refraction, to the best of our knowledge fails to address normal incidence conditions in positive phase distribution and its underlying consequences. Normal-incidence conditions can be angle-tuned by modifying the vales of the phase distribution gradients. Furthermore, for configurations around the normal-incidence angles, the metasurface will exhibit a bifunctional behavior—either divergent or convergent. All these properties are essential for applications such as optical guiding in integrated optics, wave front sensing devices, polarization controllers, wave front-to-polarization converters, holographic sensors, and spatially-resolved polarization measurement.

## 1. Introduction

Almost all of modern-age optical engineering revolves around controlling and manipulating light properties during propagation, with increasing research efforts being carried out towards either high levels of electromagnetic field control in a wide frequency range and its integration in chip-based setups, from microwave to optical spectral windows. Control of the wavefront, polarization, and direction of propagation can be realized by engineering the accumulated phase of the wave as it propagates through various media or through interfaces. Furthermore, integration of optical and terahertz circuits through waveguide designs has gained increased attention over the last decade, with efforts to realize optimal coupling in fibers [1,2,3], as well as optical and quantum circuits or memories [4,5,6]. When using interfaces, it has been demonstrated [7] that a pre-designed phase distribution across the interface gives rise to a series of effects that are either new altogether or are previously known, but the conditions in which they are obtained change dramatically. A highly-employed method of obtaining custom-designed phase distribution with no equivalent in conventional optics is the use of metasurfaces [8,9,10,11,12,13,14], which are defined as structured periodic combinations of sub-wavelength sized metallic, semiconductor, or dielectric elements placed on a substrate, and which act as a scattering meta-atom for appropriately-selected frequencies of the electromagnetic field. Phase-controlling geometries range from simple polygon-shaped patches (rods, rectangles, rings) to complex architectures made of combinations of polygon-shaped patches with strict element size relations among the components [15,16]. The desired phase distribution is obtained by creating an appropriate combination between the nature of the materials used, the design of the unit cell and the size of the composing elements with respect to each other. A broad tuning response, ranging from microwave and terahertz to the mid-infrared and visible can be achieved by changing the size of the unit cell. By now, metasurfaces have been used to demonstrate both typical and exotic optical behavior with improved quality regarding dispersion [17,18,19], and tuning range [20,21]. The set of so-called exotic materials and associated properties includes non-local response metasurfaces [22], anomalous reflection and refraction structures [7,23], optical cloaking [24,25], frequency-selective surfaces [20,26] offering the potential of giant magnetic resistance [27], epsilon near-zero metasurfaces [28,29], hyperbolic metasurfaces [30,31,32], and generalized Kerker-effect structures [33,34]. In construction of the so-called meta-atoms, metallic structures of either gold, silver, or copper were initially deposited on a dielectric substrate, changing the phase structure of the interface. These architectures, however, exhibited limited phase control and high Ohmic losses, resulting in a low efficiency limit. The almost limitless possibilities of material combinations, as well as sizes and geometries allow “artificial atoms” to control any parameter of light in all spectral ranges. More recently, versions of hybrid [35,36,37,38] and all-dielectric [39,40,41] metasurfaces have been proposed, with superior qualities in terms of wave front control, transmission coefficient, and cross-polarization. Furthermore, when combined with soft materials such as liquid crystals, on-demand external control of the metacell can be obtained, resulting in addressable absorbers and switches [42,43]. From a simplified theoretical point of view, any metasurface is able to control the behavior of the transmitted and reflected beam by modifying it according to the spatial phase pattern contained in the unit cell. For a complete control of the wavefront the metasurface has to exhibit a phase distribution with phase values ranging from zero to 2π. It was shown that standard geometric shapes with a given fixed orientation do not exhibit a full-range phase distribution [12,13]. To bypass that obstacle, full-range phase metasurface unit cells contain multiple elements rotated with a certain angle with respect to each other [44,45]. This rotation induces asymmetry in the design and is usually accompanied by a certain degree of chirality, making the surface generally suited for only one polarization mode. Very recently, it has been reported that some phase distribution designs can favor multiple functionalities of the metasurface, which becomes responsive to the input field parameters (spectrum, polarization, wave vector, etc.) [46,47,48].

In this paper, we report the investigation of the possibility of achieving equal angles of incidence and emergence at non-normal incidence angles and the transition from a converging to a diverging optical system in reflection and transmission by using a rectangular metallic and/or dielectric patch metasurface configuration. Contrary to V-shaped and H-shaped antennas described in [7,23,49], which establish negative gradient surface distribution, our patch-based configuration establishes a positive, continuous distribution of phase across the interface. To distinguish our work from those mentioned above and all their derivatives, we concentrate our discussion on the effect of field transmission at non-normal incidence angles and angle-dependent bifunctional converging/diverging behavior using positive phase distributions instead of negative ones. To our knowledge, this approach has not yet been reported. For proof-of-principle considerations, here we explore the behavior of an interface that exhibits constant gradients, although generalizations to other functions depicting the phase distribution can be performed, such as lens-type phase distribution equations.

## 2. Theoretical Model and Simulation Conditions

**Figure 1:**
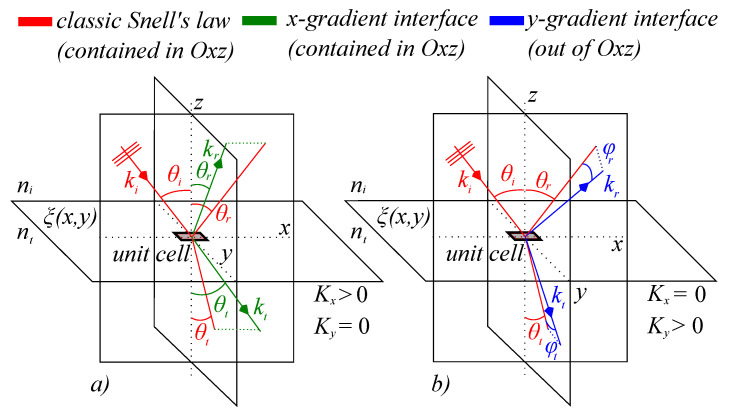
Schematic representation of general reflection and refraction on an interface with a positive-gradient phase distribution. The incident wave is considered a plane wave with a diameter comparable with the dimension of the entire interface. The incident wave is contained in the *Oxz* plane and its wave vector is depicted in solid red. In the classical, zero gradient interface case, the incident, reflected, and refracted waves are contained in the same plane *Oxz*, with reflection and refraction angles $\theta_{r}$ and $\theta_{t}$, respectively. We depict this case in solid red lines. (**a**) For a positive nonzero gradient interface with $K_{x}$ and $K_{y}$ gradients, for $K_{x}>0$ and $K_{y}=0$, the angles of the reflected and refracted waves will change, but will remain in the incidence plane *Oxz*. We depict these directions in solid green lines. (**b**) For $K_{y}>0$ and $K_{x}=0$, the reflected and refracted waves will propagate out of the incidence plane, at angles $\varphi_{r}$ and $\varphi_{t}$ with respect to the directions of the reflected and refracted classical waves. We depict this case in solid blue lines.

In research reported before [7,50], it was shown that for a wave front incident on an interface, the classical laws of reflection and refraction belong to a special case in which the phase distribution gradient across the interface is constant in the *xy* plane, assuming the interface normal is parallel to the *z* axis. To illustrate generalized reflection and refraction with positive-gradient phase distribution, we consider a wave front having a vacuum wave vector $k_{0}$ which is incident on an interface between two isotropic media with $n_{i}$ and $n_{t}$. The wave vector is in the *xz* plane and is incident on the interface at an angle $\theta_{i}$. When the interface exhibits a phase distribution given by a certain function $\xi \left(x,y\right)$, the reflected and transmitted wave vectors will no longer be in the *Oxz* plane at angles $\theta_{r}$ and $\theta_{t}$, but will deviate from said plane with angles $\varphi_{r}$ and $\varphi_{t}$, respectively. A schematic of the model is presented in Figure 1. The law of generalized reflection with respect to each axis writes as [7,23]:

(1) $$\begin{array}{c} \sin\theta_{r}-\sin\theta_{i}=\frac{1}{n_{i}k_{0}}\frac{\partial \xi }{\partial y} \end{array}$$

(2) $$\begin{array}{c} \cos\theta_{r}\sin\varphi_{r}=\frac{1}{n_{i}k_{0}}\frac{\partial \xi }{\partial x} \end{array}$$

while the law of generalized refraction can be expressed as:

(3) $$\begin{array}{c} n_{t}\sin\theta_{t}-n_{i}\sin\theta_{i}=\frac{1}{k_{0}}\frac{\partial \xi }{\partial y} \end{array}$$

(4) $$\begin{array}{c} \cos\theta_{t}\sin\varphi_{t}=\frac{1}{n_{t}k_{0}}\frac{\partial \xi }{\partial x} \end{array}$$

**Figure 2:**
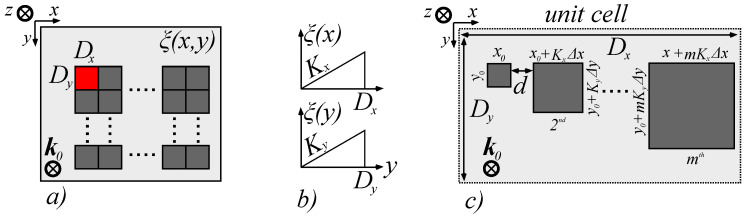
Structure of the patch-based metasurface. (**a**) Top view of the interface made of unit cells with dimensions $D_{x}$ and $D_{y}$ (pictured in red) aligned parallel to the *xy* axis set. (**b**) Schematic of the phase function along the *x* and *y* axes for one unit cell. (**c**) Top view of the unit cell implementing a constant phase gradient on *Ox*, consisting of an array of 1×m array of patches having linearly increasing sizes Δx and Δy.

The desired phase distribution $\xi \left(x,y\right)$ across the interface can be realized by depositing a periodic structure repeating along either *Ox* or *Oy*, depending on the direction we wish to achieve controllable general reflection and refraction, as presented in Figure 2a. This architecture was chosen due to the fact that the plasmonic resonance occurring along a given direction also influences the phase distribution of the surface when the wavelength deviates from said resonance, and therefore, the constant, positive phase gradients $K_{x}$ and $K_{y}$ can be encoded in such a structure. To establish a constant gradient $K_{x}$ across *Ox*, each unit cell of the structure consists of number *m* of sub-wavelength sized patches with a rectangular geometry aligned along *Ox*, in which the geometric size of the patches vary in the *xy* plane with discrete multiples *m* of Δx and Δy. This construction, however, induces a residual gradient $K_{y}$ across *Oy* which cannot be controlled, but still remains interesting from a qualitative characterization point of view. To construct the same gradient across *Oy*, we transpose the initial array to obtain a m×1 array with the same spacing *d* between components. This, however, induces a parasitic gradient across *Ox*, which has to be quantitatively characterized. The linear phase distribution is presented in Figure 2b, and the metasurface structure that realizes this distribution is presented in Figure 2c. To minimize diffraction effects arising from the periodicity of the patches, the spacing between them, *d*, must satisfy the condition d≪λ, where λ is the operation wavelength. Furthermore, to minimize diffraction effects resulting from the unit cell periodicity, the number *m* of elements has to be appropriately chosen so that the period *D* of the repeating phase distribution obtained from the number of patches *m* and individual dimensions *x* and *y* have been chosen to satisfy the relation D≥10λ. This condition is a rough estimate results from the general convention that renders diffraction effects negligible whenever the obstacle size becomes much larger than the wavelength. At the other end of the size interval, to preserve the metasurface behavior, the individual component size has to be smaller than λ/2 [51], which leads to a lower limit of m≃20. The materials used to design such patches are chosen based on the working wavelength of the application: in order to maintain a positive phase distribution gradient, the permittivity of the material has to be positive at λ, assuming the material is non-magnetic. For GHz spectra, metallic patches can be implemented without significant loss, while for THz and optical spectra, silicon and dielectric patches ensure a low-absorption interface. It can be observed that the phase gradient of the surface generates customized generalized reflection and refraction, with the following consequence: *equal angles of incidence and emergence obtained for non-normal incidence angles*. To highlight this behavior, we have considered a phase distribution $\xi \left(x,y\right)$ made out of just one unit cell with m≃20 elements aligned either in the 1×m ( parallel to *Ox*) or in the m×1 array configuration (parallel to *Oy*). By theoretically superimposing the two alignment configurations, we obtain a constant gradient of the form:

(5) $$\xi \left(x,y\right)=K_{x}x+K_{y}y$$

The magnitudes of the gradients across *Ox* and *Oy* were engineered by designing the size of the metasurface unit cell components Δx and Δy across the two axes, as illustrated in Figure 2c. Furthermore, for $K_{x},K_{y}>0$, we show that there are configurations that permit tuning as to obtain equal angles of incidence and emergence at non-normal incidence angles. For other angles not belonging to this tuning curve, the behavior of the metasurface is either converging or diverging, leading to a bifunctional structure. Based on the model described above, we have evaluated the values of the direction angles $\theta_{t,r}$ and $\varphi_{t,r}$ for the reflected and transmitted waves, and we have highlighted the changes in behavior with respect to the zero phase gradient interfaces known from classical optics.

Theoretical investigations were carried out using two commercially-available software systems: Simulations were conducted with the aid of COMSOL Multiphysics–radio frequency (RF) module, and data processing was conducted using Matlab. From a simulation framework perspective, we have chosen an interface between air as the incident medium, and standard glass ($n_{t}≃1.52$, no loss) as the transmission medium. The input field was chosen to be plane-wave, with a working wavelength λ=1.5m, corresponding to a frequency f=200 THz. The electric field is parallel to the *Ox* axis, while the magnetic field and wave vector are contained in the *yz* plane, and linked to the specific incidence angle. In each unit cell, the first element of the array has a square shape $x_{0}=y_{0}=100$ nm, and the spatial increment unit was Δx=Δy=20 nm. The distance between the cells is d=500 nm. Copper with a conductivity σ≃58.7 S/m was chosen as construction material for the metallic patches. The thickness of the patches is considered negligible, as in practice the interface has subwavelength-thick elements deposited on the glass substrate. Using these design considerations, we have investigated the behavior of both in-plane and out-of-plane transmission and reflection angles, as well as their associated phases as a function of incident angle and ratio between the gradient magnitude (both $K_{x}$ and $K_{y}$) and the vacuum wave vector $k_{0}$, taken over one equivalent spatial cycle. The values of both spatial gradients were varied from zero to $2k_{0}$. The simulation solves the spatial component of the wave equation relying on a FDTD technique in which the resolution of the mesh element is below λ/50. To highlight the particularities of our study, the boundary conditions at the interface have to be modified in order to introduce the effects described by Equations (1)–(4) in the form discussed above. The periodic structure was modeled by Floquet periodicity, with the wave vector computed numerically directly by the simulation environment. We reduced the study to one unit cell instead of the full interface to reduce computation time and to trace out parasitic diffraction effects that inevitably appear when a periodic structure is involved. Under all these considerations, the simulation was set to determine the directions of the transmitted and reflected wavefronts after the interaction with the interface, assuming the modeled phase gradients, at a fixed wavelength. The directions were calculated as a result of the phase matching principle and the specific boundary conditions imposed at the interface. These directions were further processed to calculate the in-plane and out-of plane angles that define the propagation of the reflected and transmitted waves.

## 3. Results and Discussions

**Figure 3:**
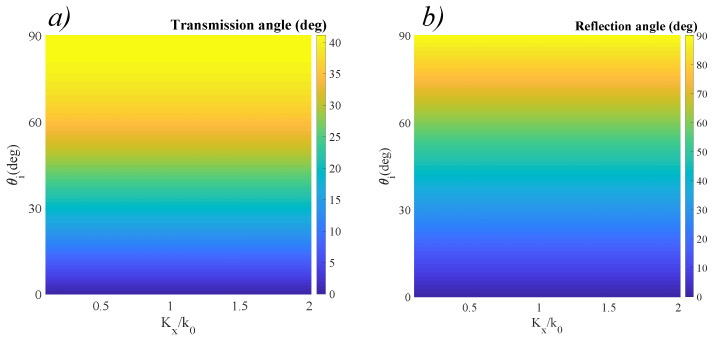
(**a**) Transmission angle $\theta_{t}$, and (**b**) reflection angle $\theta_{r}$ obtained for a zero-gradient metasurface in the air-to-glass incidence scenario ($n_{t}≃1.52$). The gradient is included both to show there is no influence of the interface on the working wavelength, and to serve as a reference for the other cases.

To better illustrate the significant change in behavior, as well as to test our simulation setup, we first assumed a classic, zero-phase-map interface between air and glass, and we represented the classic laws of reflection and refraction in two dimensional color maps. We represent the classic case of reflection and refraction as a function of gradient even if there is no gradient available, to enhance the visibility of the modified behavior once the gradients are introduced. These results are presented in Figure 3.

**Figure 4:**
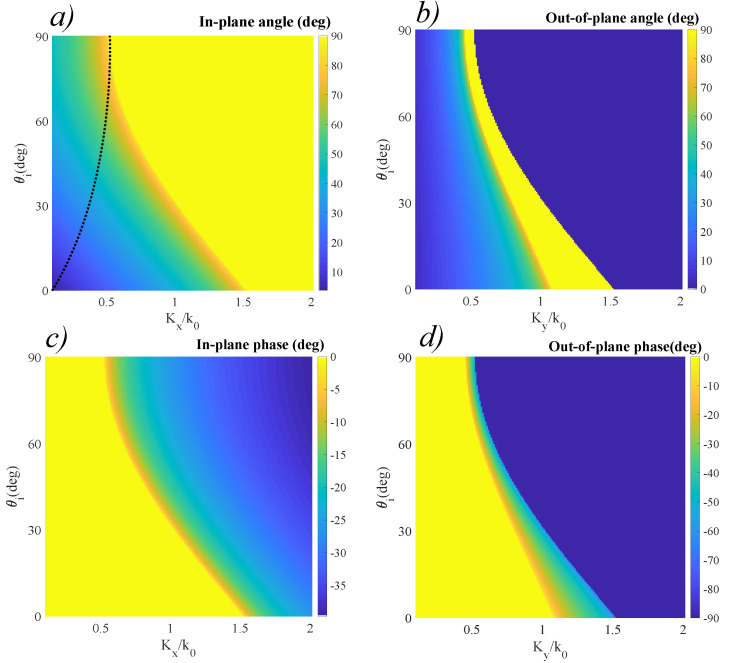
Transmission properties of a linear-gradient optical interface. (**a**) In-plane transmission angle $\theta_{t}$ obtained for a metasurface with a constant positive gradient $K_{x}$ across the *Ox* direction. The dotted line represents the tuning curve to achieve equal angles of incidence and emergence at non-normal angles. (**b**) Out-of-plane transmission angle $\varphi_{t}$ for a metasurface with a positive linear gradient $K_{y}$ across *Oy*. (**c**) Associated phase of the transmitted wave for the $K_{x}—$defined metasurface. (**d**) Associated phase of the transmitted wave for the $K_{y}—$defined metasurface.

Assuming no dispersion effects, the zero-phase interface is wavelength-independent across a broad spectrum (i.e., the same refraction angle is obtained at any ratio), although the resonant properties of the surface will modify the amplitudes of the reflected and refracted waves. As expected from theory, both reflected and refracted waves are contained in the incidence plane, defined as the plane containing the incident wave and the normal to the surface. Since $n_{i}<n_{t}$, there is no critical angle, and the phase between the transmitted and incident waves remains zero. These conditions are clearly represented by the results obtained in Figure 3. Moreover, the phase of the transmitted wave is zero, while for the reflected angle it is π, for all incident angles. We now modify the phase distribution of the surface as to exhibit constant positive linear gradients $K_{x}$ and $K_{y}$ across *Ox* and *Oy*, with values that are comparable to the wave vector associated to the working wavelength. This scheme can be modified to serve at any desired wavelength, by appropriate modifications of the materials used and geometric dimensions. The results that were obtained from reapplying the model equations for general refraction as boundary conditions to the air-metasurface interface in the conditions described at the previous section are presented in Figure 4. In this case, the designed phase distribution introduces a significant change in the transmission response. Given $n_{i}<n_{t}$, a constant $K_{x}$ and $K_{y}=0$, we can observe a bifunctional behavior of the interface: for low incidence angles, the metasurface acts as a diverging lens. In the special case of normal incidence, the transmitted wave respects normal incidence only for $K_{x}=0$. For nonzero values of $K_{x}$, we can see that $\theta_{t}>\theta_{i}$. This leads to a series of directional wave guiding applications that have the normal incidence constraint embedded in their characteristic. For a non-normal incidence angle, when $K_{x}=0$, we obtain the classical refraction case (i.e., a converging surface). As $K_{x}$ increases, the angle $\theta_{t}$ becomes greater than $\theta_{i}$, implying that the behavior of the surface becomes diverging. The behavior is continuous, which implies that there is always a certain inflection angle $\theta_{0}$ which is *direction-preserving*. This special configuration between the incident angle and spatial phase gradient $K_{x}$ that ensures equal angles of incidence and emergence between the incident and in-plane wave is represented by the dotted line in Figure 4a. The existence of this configuration implies that, for a specially-designed phase gradient interface, the optic axis can be artificially designed. Such designer surfaces have ideal applications as broad-angle optical couplers for fiber optics.

The presence of a non-zero phase gradient allows the possibility of obtaining a critical angle even for $n_{i}<n_{t}$. In the case of normal incidence, we can obtain a critical angle for a value $K_{x}>1.5$. Furthermore, by setting $K_{y}=C\neq 0$, out-of-plane refraction can be obtained across a full-angle spectrum. These conditions are represented in Figure 4b. When considering normal incidence, the out-of-plane angle $\varphi_{t}$ varies from zero, for $K_{x}=0$, which corresponds to the classical case, to $90^{\circ }$, for $K_{x}/k_{0}≃1.5$. For values $K_{x}/k_{0}>1.5$, the angle abruptly shifts back to zero. For non-zero incidence angles, the behavior of the surface changes, but maintains the same tendency of continuous increase, followed by an abrupt shift to zero. This abrupt switching behavior can be exploited in the fabrication of high quality switches and waveguides, able to send part of the field in a direction that does not experience the in-plane angle wave. Simultaneously, the phase induced by the phase gradient metasurface changes significantly with respect to the classic case introducing a phase difference between the transmitted and incident waves, as indicated in Figure 4c,d. Contrary to the switching behavior exhibited by the out-of-plane angle, the phase accumulated by the reflected and transmitted waves varies continuously between zero and $40^{\circ }$ for the in-plane wave phase, and between zero and $90^{\circ }$ for the out-of-plane wave phase. This permits a partial control on the polarization of the two waves, with the possibility of obtaining elliptical polarization and, in the case of the out-of-plane wave, even circular polarization.

**Figure 5:**
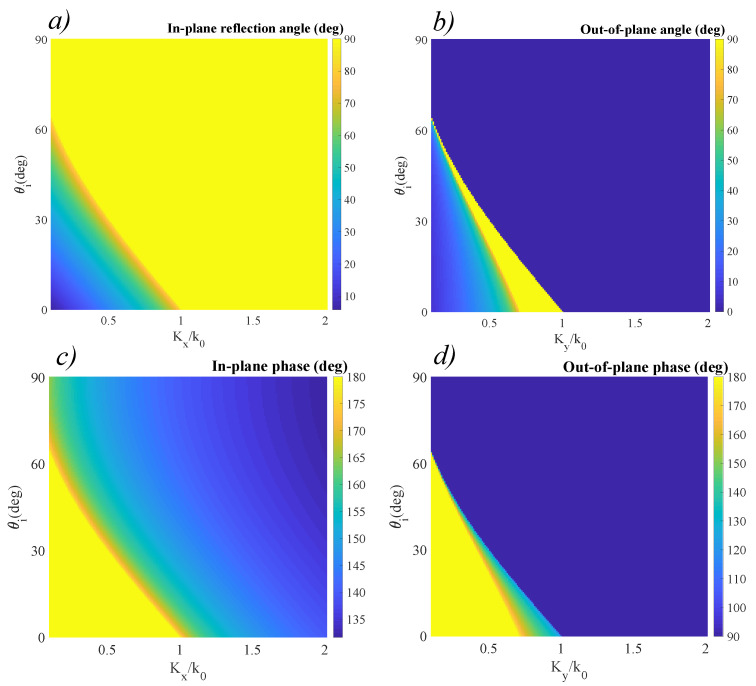
Reflection behavior of a linear-gradient optical interface. (**a**) In-plane reflection angle $\theta_{r}$ obtained for a metasurface with a constant positive gradient $K_{x}$ across *Ox*. (**b**) The associated phase of the reflected in-plane wave. (**c**) Out-of plane reflection angle $\phi_{r}$ for a metasurface with a constant positive gradient $K_{y}$ across *Oy*. (**d**) The associated phase of the out-of-plane wave.

The data obtained for the reflected waves using the same conditions as detailed above is presented in Figure 5. The positive gradients induce an increase in the values of the in-plane reflected angle, as highlighted in Figure 5a. Again, considering the absence of phase gradients, the incidence angle respects the classic configuration, in which $\theta_{r}=\theta_{i}$ for the in-plane wave. As the values of the phase gradients increase, the reflected angle increases, until a critical angle is achieved. For normal incidence, this critical angle is achieved at $K_{x}/k_{0}≃1$. As the angle of incidence increases, the critical angle is obtained for lower values of $K_{x}/k_{0}$. This increase allows the achieving of critical reflection at lower angles than in the classic case by appropriate gradient selection. We also observe the appearance of out-of-plane reflection when $K_{y}$ is nonzero, as indicated in Figure 5b. Again, for normal incidence we can observe that for values $K_{y}/k_{0}$ between 0.7 and 1, a critical angle is obtained. This behavior is followed by a sharp cutoff, at which the direction shifts to $0^{\circ }$. For some configurations of non-normal incidence angles and nonzero $K_{y}$, the transition from zero to $90^{\circ }$ is increasingly abrupt, and for incidence angles exceeding $60^{\circ }$ the out-of-plane component is no longer observed. The associated phases are no longer constant, but follow a bi-dimensional dependency, as indicated in Figure 5c,d. Both phases follow a continuous distribution, ranging from π, in the classic configuration to $135^{\circ }$ for the in-plane wave phase, and $90^{\circ }$ for the out-of plane wave phase. Furthermore, in the case of the out-of-plane wave phase, for nonzero incidence angles and nonzero $K_{y}$, the transition from $180^{\circ }$ to $90^{\circ }$ takes place with increased sharpness. All these considerations allow partial control of the polarization from linear to elliptic in the case of the in-plane wave and from linear to circular in the case of the out-of-plane wave, respectively.

## 4. Conclusions

In this paper, we report theoretical conditions of achieving equal angles incidence and emergence at non-normal incidence angles using positive-phase distribution metasurfaces, coupled with a bifunctional behavior of such structures. We performed investigations of the modified behavior and referenced them to the classical Snell laws of reflection and refraction. The data show that given a positive phase distribution, there exists a so-called normal incidence tuning curve, which can be obtained at non-normal incidence angles and appropriately-designed phase distribution. Moreover, an out-of-plane phase gradient induces out-of-plane reflection and refraction components. Both in-plane and out-of-plane components exhibit variable phases, leading to controllable polarization both in transmission and reflection. We believe that the proposed model is scalable from GHz to optical spectral regions by adjusting the geometric dimensions of the metasurface elements or the materials used in the construction of the structure. These types of designed interfaces permit enhanced control of the wave front and polarization of input electromagnetic waves, with applications ranging from non-normal incidence light guiding to light trapping at near-normal incidence and interface-controlled polarization devices.

## Data Availability

The data presented in this study are available on request from the corresponding author.
